# The Relationship of Pyroptosis-Related Genes, Patient Outcomes, and Tumor-Infiltrating Cells in Bladder Urothelial Carcinoma (BLCA)

**DOI:** 10.3389/fphar.2022.930951

**Published:** 2022-07-19

**Authors:** Ruiyan Xie, Ming Xie, Litong Zhu, Joanne W. Y. Chiu, Wayne Lam, Desmond Y. H. Yap

**Affiliations:** ^1^ Division of Nephrology, Department of Medicine, Queen Mary Hospital, The University of Hong Kong, Hong Kong, Hong Kong SAR, China; ^2^ Department of Urology, Nanfang Hospital, Southern Medical University, Guangzhou, China; ^3^ Division of Haematology, Medical Oncology and Haemopoietic Stem Cell Transplantation, Department of Medicine, Queen Mary Hospital, The University of Hong Kong, Hong Kong, Hong Kong SAR, China; ^4^ Division of Urology, Department of Surgery, Queen Mary Hospital, The University of Hong Kong, Hong Kong, Hong Kong SAR, China

**Keywords:** pyrotosis, CASP8, immune cells, bladder urothelial carcinoma, immunotherapy

## Abstract

**Introduction:** The role of pyroptosis and its effects on tumor-infiltrating cells (TICs) in the pathogenesis and treatment outcomes of patients with bladder urothelial carcinoma (BLCA) remains unclear.

**Methods:** We conducted a bioinformatics analysis on the pyroptosis-related genes (PRGs) and TICs using data from public domains, and evaluated their impact on the pathogenesis and clinical outcomes of BLCA patients. A risk score based on PRGs and a prognostic risk model that incorporated patient demographics, tumor characteristics, and differentially expressed genes (DEGs) were developed.

**Results:** Twenty-three DEGs of 52 PRGs were identified in BLCA and normal samples from the TCGA database. Missense mutations and single nucleotide polymorphisms in PRGs are the most common genetic abnormalities. Patients with high PRG risk scores showed an inferior survival compared to those with low risk scores. The prognostic risk model based on patient demographics, tumor characteristics, and DEGs showed good predictive values for patient survival at 1, 3, and 5 years in BLCA patients. Caspase-8 (CASP8) was the only intersection gene of the prognostic genes, DEGs, and different genes expressed in tissue. Patients with a high CASP8 expression had improved survival, and an increased CASP8 expression level was observed in activated CD4 memory T cells, follicular T helper cells, resting NK cells, M0 macrophages, and activated dendritic cells. CASP8 expression also showed a positive correlation with the IL7R expression—a key cell marker of CD4 memory T cells. CASP8 expression also showed correlations with immune checkpoints (PDCD1, CD274, and CTLA4) and response to immune checkpoint inhibitors.

**Conclusion:** Our data suggest that PRGs, especially CASP8, showed strong associations with patient outcomes and TICs in BLCA. If validated, these results could potentially aid in the prognostication and guide treatment in BLCA patients.

## Introduction

Bladder urothelial carcinoma (BLCA) is a common urological malignancy worldwide, with approximately 5,50,000 new cases each year (Richters et al., 2020). Over 90% of the bladder cancers are urothelial carcinomas of the bladder (UCB). The management of UCB has evolved substantially over the past few decades.

Approximately, 70% of patients are present with non-muscle invasive bladder cancer (NMIBC), which is typically treated with transurethral resection of bladder tumor (TURBT) and subsequent intravesical chemotherapy or immunotherapy in intermediate- to high-risk groups. However, they are associated with high recurrence and disease progression rates. Up to 80% of the patients presented with NMIBC relapse within 5 years of diagnosis, with 30% of the patients showing disease progression. The remaining 30% of the patients are present with muscle invasive bladder cancer (MIBC) (Zhu et al., 2019). For these groups of patients, if the disease remains localized, the recommended treatment options that should be considered include radical cystectomy with urinary diversion and radical radiotherapy, and neoadjuvant and/or adjuvant chemotherapy or immunotherapy (Cathomas et al., 2022). For those with advanced metastatic UCB, cisplatin-containing chemotherapy have been shown to improve overall survival (OS), while immunotherapy using PD-1/PD-L1 checkpoint inhibitors have shown promising initial results (Powles et al., 2019).

These treatments, however, are not without significant surgical risks and treatment-related toxicities. Despite all these progresses in therapeutics, patients with advanced or invasive BLCA still have significant morbidities and unfavorable clinical outcomes. There are currently no tools available to facilitate the selection of patients who have a higher probability of benefitting from chemotherapy or immunotherapy for these groups of patients. Therefore, novel biomarkers that can serve as therapeutic targets or prognostic indicators are eagerly awaited to help differentiate responders from non-responders.

The pathogenesis of BLCA is highly complex. Putative pathogenic mechanisms include chronic infection, epigenetic alterations, DNA methylation, etc. (Hatta et al., 2021). Pyroptosis is a crucial form of programmed cell death that participates in the host’s immune defense against different pathogens (D’Souza and Heitman, 2001). Pyrotopsis is triggered by inflammasomes upon the encounter of intracellular danger signals, and is characterized by the activation of cascades of caspases different from those in apoptosis (Bergsbaken et al., 2009). Dysregulation of pyrotosis has been proposed as a crucial mechanism for carcinogenesis. For example, the deficiency of GSDME, a pyroptosis-related gene, was reported to suppress the activity of melanoma cells (Rogers et al., 2019). Similar results were found in breast cancer, colorectal cancer, gastric cancer, and hepatocellular carcinoma (Yu et al., 2021). Reduced pyroptosis-associated inflammasomes such as AIM2, NLRP1, and NLRC4 were observed in a colorectal cancer mice model (Karki et al., 2017). However, the role of pyroptosis in BLCA remains poorly understood. Another important pathogenic mechanism pertinent to cancer development and progression is the tumor-microenvironment (TME) and tumor infiltrating cells (TICs). Aberrations in the immune surveillance have contributed to the development, progression, and metastatic potential of cancers. Various immune cells such as macrophages, dendritic cells (DC), natural killer (NK) cells, as well as B and T lymphocytes have all be implicated in the immune surveillance of neoplastic conditions (Hinshaw and Shevde, 2019), and it is well recognized that each type of TICs has specific signatures that determine how the cancer affects neighboring cells and survives (Wang et al., 2018). A better understanding on the TME and TICs in BLCA is clinically important because of the growing application of immunotherapy in combating this neoplasm and cell-based immunotherapies have also emerged as novel and promising therapeutic strategies for BLCA (Morales and Paramio, 2021). However, the interaction between pyroptosis and immune cells remain poorly understood.

As such, we conducted a bioinformatics analysis on pyroptosis-related genes (PRGs) and TICs using data from public domains, and examined their impact on the pathogenesis and clinical outcomes of patients with BLCA. These results may elucidate new therapeutic mechanisms of existing and novel therapies and help select suitable patients for different treatment modalities.

## Materials and Methods

### Dataset Collection

The BLCA RNA sequencing (RNA-seq) data (normal count: 19 cases; tumor count: 414 cases) and the copy number variation (CNV) data of BLCA were obtained from The Cancer Genome Atlas (TCGA) database (https://portal.gdc.cancer.gov/) and the external validation cohort (GSE19423, 48 cases) was obtained from the Gene Expression Omnibus (GEO) (https://www.ncbi.nlm.nih.gov/geo/). Fifty-two PRGs were selected from prior publication (Song et al., 2021) and the MSigDB database (http://www.broad.mit.edu/gsea/msigdb/).

### Mutation Analysis of PRGs

The analysis of the mutation frequency and the classification of 52 PRGs were performed by the *R* package “maftools.” The location of CNV alteration of the PRGs on the 23 chromosomes was assessed by “RCircos” *R* package.

### Identification of Differentially Expressed Genes

The *R* package, limma (Law et al., 2016), was employed to analyze differentially expressed genes (DEGs) with thresholds of false discovery rate (FDR) = 0.05 and | log2FC| = 1. The correction network of DEGs and PRGs was generated by the “igraph” (Auer et al., 2018) *R* package.

### Classification of BLCA Subtypes Based on PRGs and DEGs

An unsupervised consensus *k*-means clustering analysis was utilized to classify BLCA patients into PRG clusters on the basis of PRG regulators’ prognostic expression. When the clustering variable (*k*) was increased from 2 to 9, *k* = 2 was identified by the consensus clustering algorithm (Kaarik and Parna, 2009). Thus, BLCA cases were divided into two subtypes, namely Subtype A and Subtype B. The relationship of the two subtypes (A = 250 cases, B = 206 cases) were evaluated by the principal component analysis (PCA). To further elucidate the biological characteristics of PRG-related BLCA molecular subtypes, DEGs in both A and B subtypes were analyzed. Adjusted *p* values of <0.05 and |log2FC| > 1 were considered as statistically significant. We also used the “ConsensuClusterPlus” *R* package to stratify patients into three DEGclusters (A = 138 cases, B = 101 cases, C = 217 cases) according to the expression of prognostic DEGs.

### Analysis of Overall Survival (OS)

The survival data of 456 BLCA cases (408 patients from TCGA with detailed clinical data and 48 patients from the GSE19423 cohort) were analyzed using the “survminer” *R* package. The survival of different BLCA subtypes were compared by a log-rank test according to the Kaplan–Meier method, and a *p* value <0.05 was considered statistically significant.

### Construction of a PRG Prognostic Risk Model for BLCA

Four hundred and fifty-six BLCA patients were randomized into two cohorts: a training cohort (*n* = 228) and a testing cohort (*n* = 228) using the “caret” *R* package in a 1:1 ratio. The training cohort was utilized for prognostic risk model building, while the testing cohort was used to validate the model. The performance of the prognostic risk model was evaluated again using patients from the entire cohort (*n* = 456). Furthermore, the LASSO–Cox regression algorithm was employed to screen out the representative genes based on the minimum criteria of cross-validation. The BLCA risk prognostic model was constructed by performing a multivariate Cox regression analysis and “glmnet” *R* package. The coefficients evaluated by LASSO regression analysis and prognostic gene expression were used to obtain a prognostic risk score in the training cohort. The score calculation formula is as follows:

$$\mathrm{Risk}\;\mathrm{scores}=\sum_{i=1}^{n}\left(Expi*Coefi\right).$$ (1)

The survival probabilities of the patients were compared between those with high- or low-PRG risk scores, using the median PRG score as the cut-off. ROC curves and survival curves according to the PRG risk score status were constructed. The “rms,” “regplot” packages were used to generate nomograms for the prediction of OS at 1, 3, and 5 years in BLCA patients and calibration plots.

### Gene Set Enrichment Analysis

The pathway enrichment analysis was carried out with the GSEA software and the target set was utilized to identify the underlying function with thresholds of *p* < 0.05. The biological functions, such as TNM stage, were assessed by the “heatmap” package.

### CIBERSORT Analysis

The CIBERSORT analysis was applied to estimate the proportions of tumor infiltrating immune cells (TICs) in tumor cases and *p*-values <0.05 were considered statistically significant.

### Assessment of the Relationship Between Target Genes and Cell Surface Markers of Immune Cells

The data on the cell surface markers of circulating immune cells were obtained from the CellMarker website (http://bio-bigdata.hrbmu.edu.cn/CellMarker/). Further relationship between CASP8 and immune cells markers were examined using the Gene Expression Profiling Interactive Analysis (GEPIA) (http://gepia.cancer-pku.cn/index.html) and Tumor Immune Estimation Resource (TIMER) (http://timer.cistrome.org/).

### Analysis of Therapeutic Sensitivity

The concentration inducing 50% reduction growth (IC50) of the targeted inhibitors (TIs) was assessed by the “pRRophetic” *R* package (Geeleher et al., 2014). Also, the association of CASP8 with immune checkpoints was explored using the GEPIA database. Moreover, the Immunophenoscores (IPS) data of BLCA patients in The Cancer Immunome Atlas (TCIA) database (https://tcia.at/home) was using to predict the immunotherapy effect based on “ggpubr” “limma” *R* package.

## Results

### Identification of PRG Genetic Variation in BLCA Patients

**FIGURE 1:**
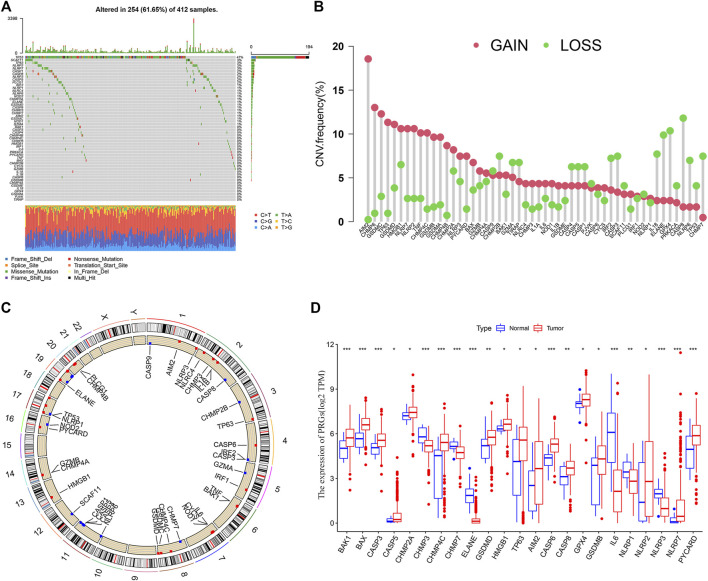
Landscape of genetic and transcriptional alterations in bladder urothelial carcinoma. **(A)** Mutation frequency of 52 PRGs using the TCGA cohort. **(B)** CNV Frequency of 52 PRGs with gain or loss. **(C)** Locations of CNV alteration in PRGs on 23 chromosomes. **(D)** The transcriptional expression of differentially expressed genes (DEGs). *p < 0.05, **p < 0.01, and ***p < 0.001.

BLCA patients from the TCGA cohort showed a high PRG-related mutation frequency (61.65%) (Figure 1A). Of all the BLCA cases, TP53 had the highest mutation frequency (47%), followed by SCAF11, TP63, NLRP7, CASP1, CASP8, NLRP3, CASP5, and PLCG1. The most common mutated classification is missense mutation and single nucleotide polymorphisms (SNPs) ranked as the top variant type (T > A being the most common substitution). Since copy number alterations (CNV) are linked to metastasis and may indicate a poor prognosis (Yu et al., 2017), we evaluated the CNV frequency of PRGs in BLCA cases. CASP8, ELANE, and GPX4 showed extensive CNV reduction while AIM2, CHMP6, and GSDMC exhibited significant CNV gain (Figure 1B). The locations of the CNV alterations of PRGs in the chromosomes are presented in Figure 1C. We further analyzed the transcriptional levels of the PRG members in BLCA (Figure 1D). Compared with the normal subjects, there is a significant overexpression in AIM2, TP63, and CHMP4C (all with CNV gain) but down-regulation of ELANE and CHMP7 (both with CNV loss). While GSDMB, NLRP7, and PYCARD were associated with CNV gain, they showed reduced mRNA expressions.

### Identification of PRG Subtypes in BLCA

**FIGURE 2:**
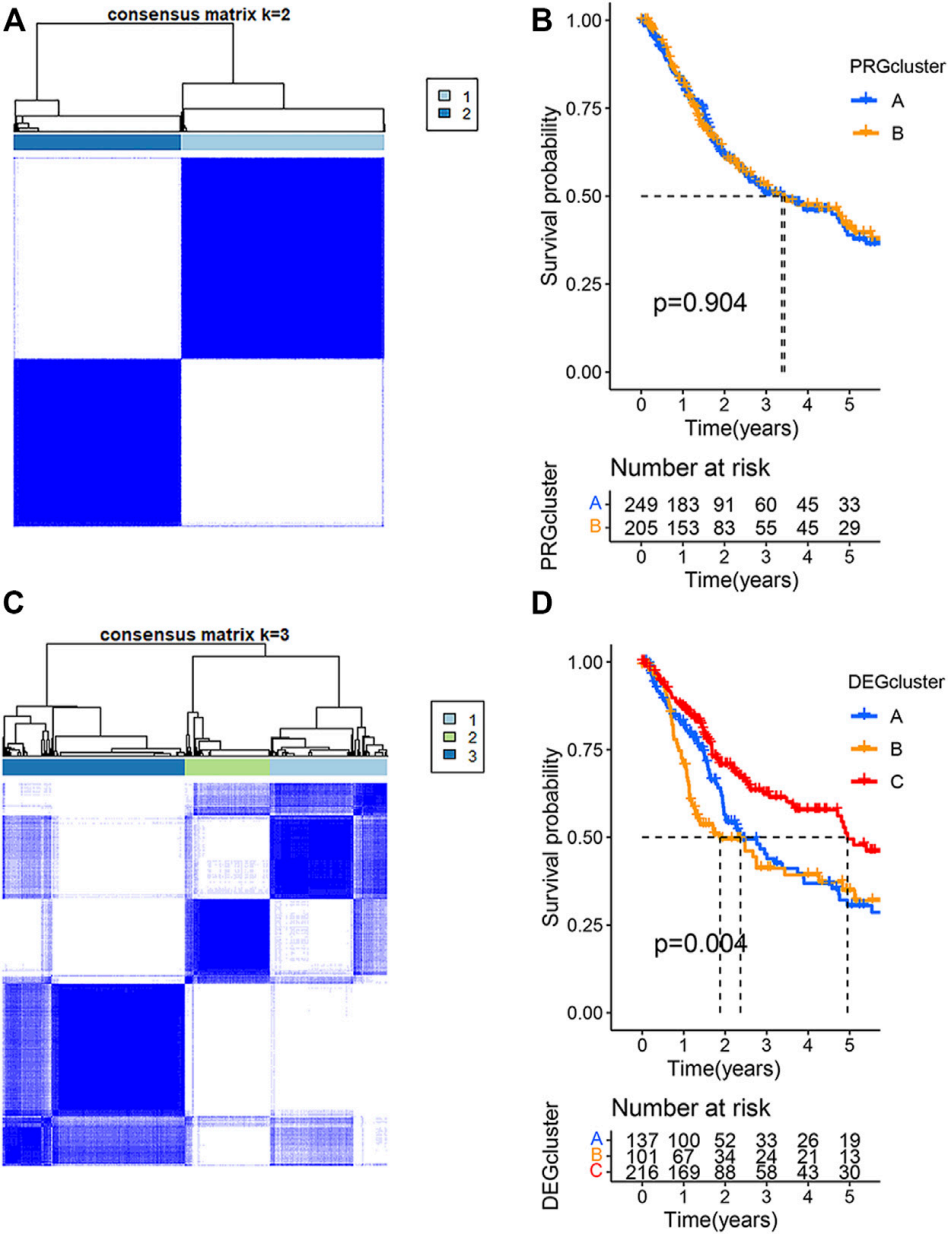
Clustering analysis of pyroptosis-related genes (PRGs) in bladder urothelial carcinoma using the TCGA and GSE19423 cohorts. **(A)** The consensus clustering algorithm showed that the optimal number of clusters was two based on the PRG expressions. **(B)** The survival analysis of two PRGcluster subtypes using Kaplan–Meier curves. **(C)** The optimal number of clusters was three based on the expression of two PRGcluster subtype- related differentially expressed genes (DEGs). **(D)** Survival analysis of three DEGcluster subtypes using Kaplan–Meier curves.

To understand the relationship between the clinical characteristics of the PRG expression and BLCA, the TCGA-BLCA cohort and GSE19423 cohort were merged for a subsequent analysis. BCLA patients were categorized into PRG Subtype A (*n* = 250) or Subtype B (206) based on the PRG expression using a consensus clustering algorithm (Figure 2A). There was no significant survival difference between the two PRG subtypes (*p* = 0.904) (Figure 2B). To better apply PRG subtypes for BLCA clinical treatment, BLCA patients were further categorized into DEGcluster Subgroups A (138 patients), B (101 patients), and C (217 patients) based on the expressions of subgroup-related DGEs using a consensus clustering algorithm. 1,792 intersecting subtype DEGs were identified with a value filter of *p* < 0.05 and *q* < 0.05 using the consensus clustering algorithm. Our result showed that when *k* = 3, DEGcluster molecular subtypes (A = 138 cases, B = 101 cases, and C = 217 cases) appeared to be the optimal one (Figure 2C). DEGcluster C was associated with a better 5-year survival rate than DEGcluster A and B (*p* = 0.004) (Figure 2D). Pyroptosis-associated DGEs such as CASP1, GZMA, and NLRC4 were significantly down-regulated in the DEGcluster C.

### Development and Validation of the PRG Score

**FIGURE 3:**
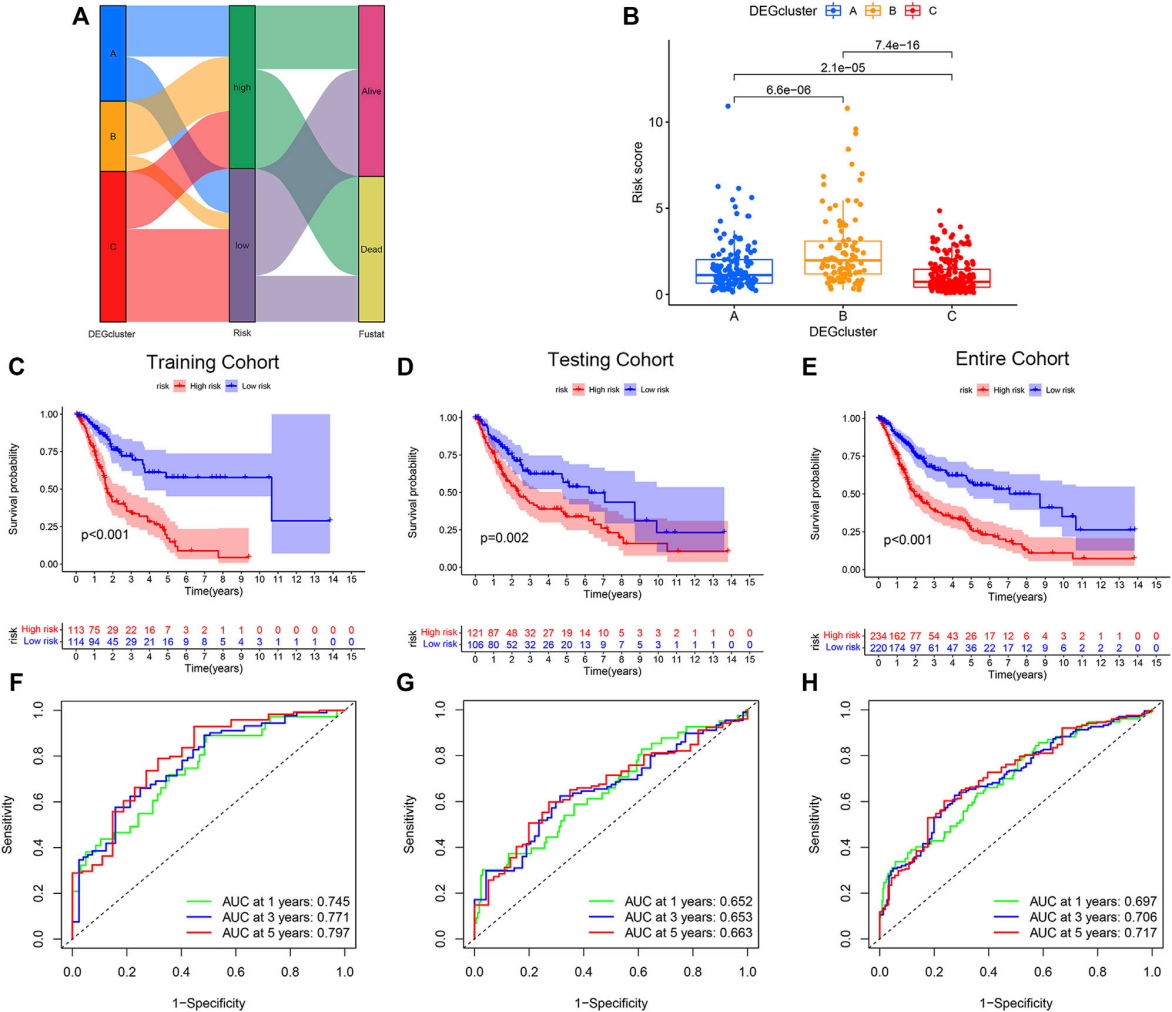
Construction and validation of the pyroptosis-related genes (PRGs) risk score model. **(A)** Sankey diagram of the bladder urothelial carcinoma risk score model constructed network. **(B)** Comparison of the risk scores of DEGcluster subtypes. Overall survival of patients with high- or low-PRG risk scores in the **(C)** training, **(D)** testing, and **(E)** the entire cohort. Receiver operator characteristics (ROC) curves of the risk score models for the **(F)** training, **(G)** testing, and **(H)** the entire cohorts.

To further apply PRG subtypes for personalization of BLCA treatment, we developed a pyroptosis-related signature score (PRG score) based on subtype-associated DEGs. Seven pyroptosis-associated genes were first selected as an independent prognostic factor by LASSO and multivariate Cox regression analyses in the training cohort. The proposed PRG risk score= (0.3032 × expression value of PVR) + (0.1427 × expression value of EMP1) − (0.5570 × expression value of APOL6) − (0.2868 × expression value of SH2D2A) − (0.2969 × expression value of TTLL3) − (0.1931 × expression value of TSPAN8) − (0.1270 × expression value of SP6). The construction process of the PRG score is shown in Figure 3A. Patients in the DEGcluster C showed significantly lower risk scores compared to DEGclusters A and B (*p* = 6.6e^−06^, 7.4e^−16^, respectively) (Figure 3B).

BLCA patients with high-PRG risk scores showed inferior survival compared with patients with low-PRG risk scores (*p* < 0.01, for all), and the results were consistent across training, testing, and the entire cohort (Figures 3C–E). The PRG risk scores were predictive of a patient’s survival at 1, 3, and 5 years in the training cohort (AUC ROC: 0.745, 0.771, and 0.797 for 1, 3, and 5 years, respectively) (Figure 3D). The AUC ROC for predicting patient survival at 1, 3, and 5 years by the PRG risk scores in the testing and entire cohort were similar to the training cohort (Figures 3F–H).

### Construction of a Predictive Nomogram

**FIGURE 4:**
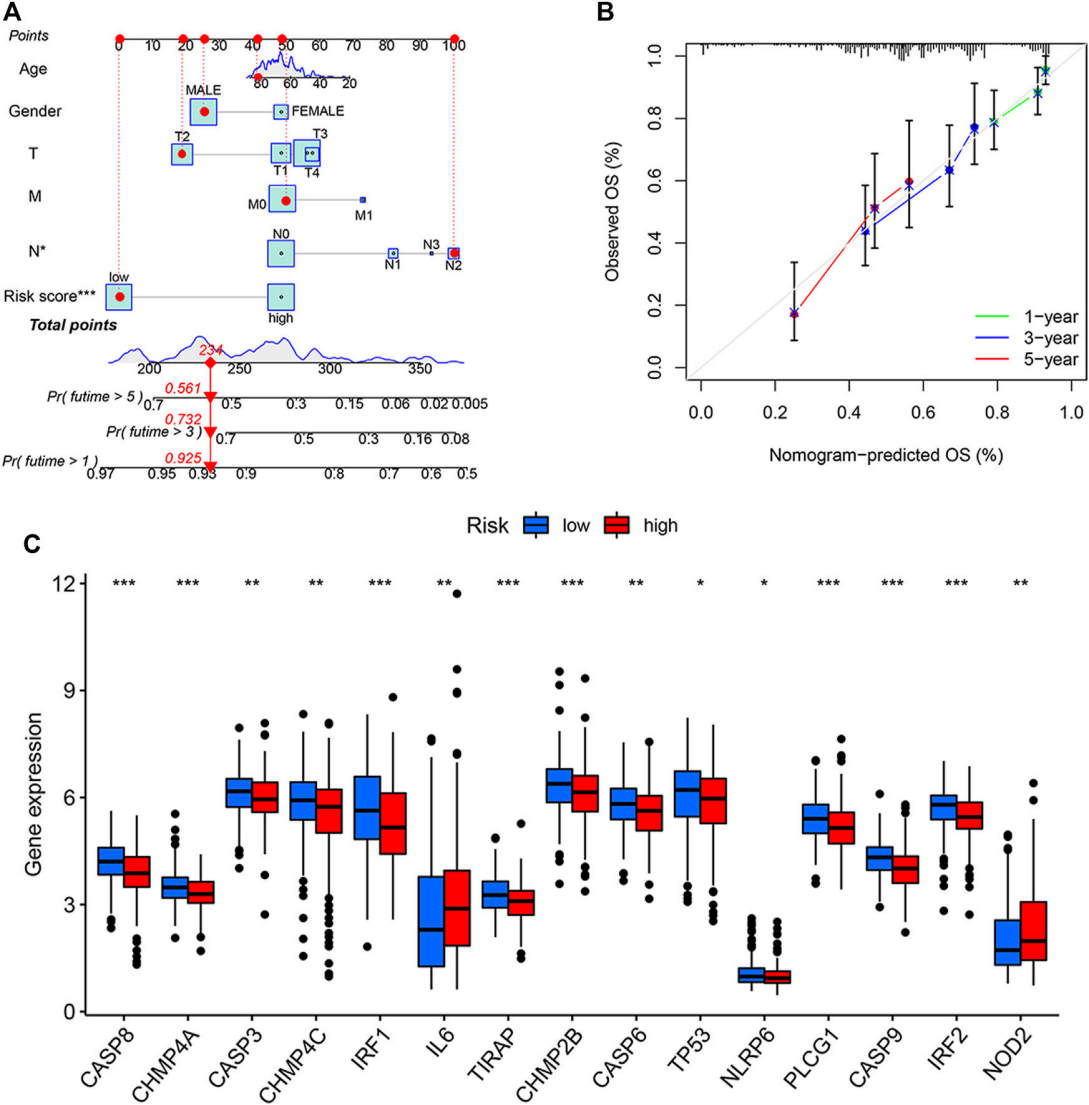
Generation and validation of a predictive nomogram. **(A)** The nomogram of the risk score and clinical characteristics including age, gender, and the TMN stage in the training cohort. **(B)** The calibration curves showed good concordance between nomogram-predicted and observed overall survival (OS). **(C)** Differentially expressed gene (DEGs) expressions of PRGs in patients with high or low pyroptosis-related gene (PRG) risk scores. *p < 0.05, **p < 0.01, and ***p < 0.001. T, tumor; M, metastases; N, nodes.

We constructed a nomogram that incorporated age, gender, TMN, and PRG risk scores to predict the overall survival (OS) at 1, 3, and 5 years in BLCA patients (Figure 4A). The nomogram-predicted OS showed good concordance with the observed OS, especially for the 1-year OS (Figure 4B). Pyroptosis DEGs associated with low/high risk are shown in Figure 2C. The patients with high risk scores showed up-regulated expressions of IL-6 and NOD2 but the down-regulation of CASP8, CHMP4A, CASP3, CHMP4C, IRF1, and TIRAP compared to the patients with low risk scores (Figure 4C).

### CASP8 Was Identified as a Novel Prognostic Biomarker in BLCA

**FIGURE 5:**
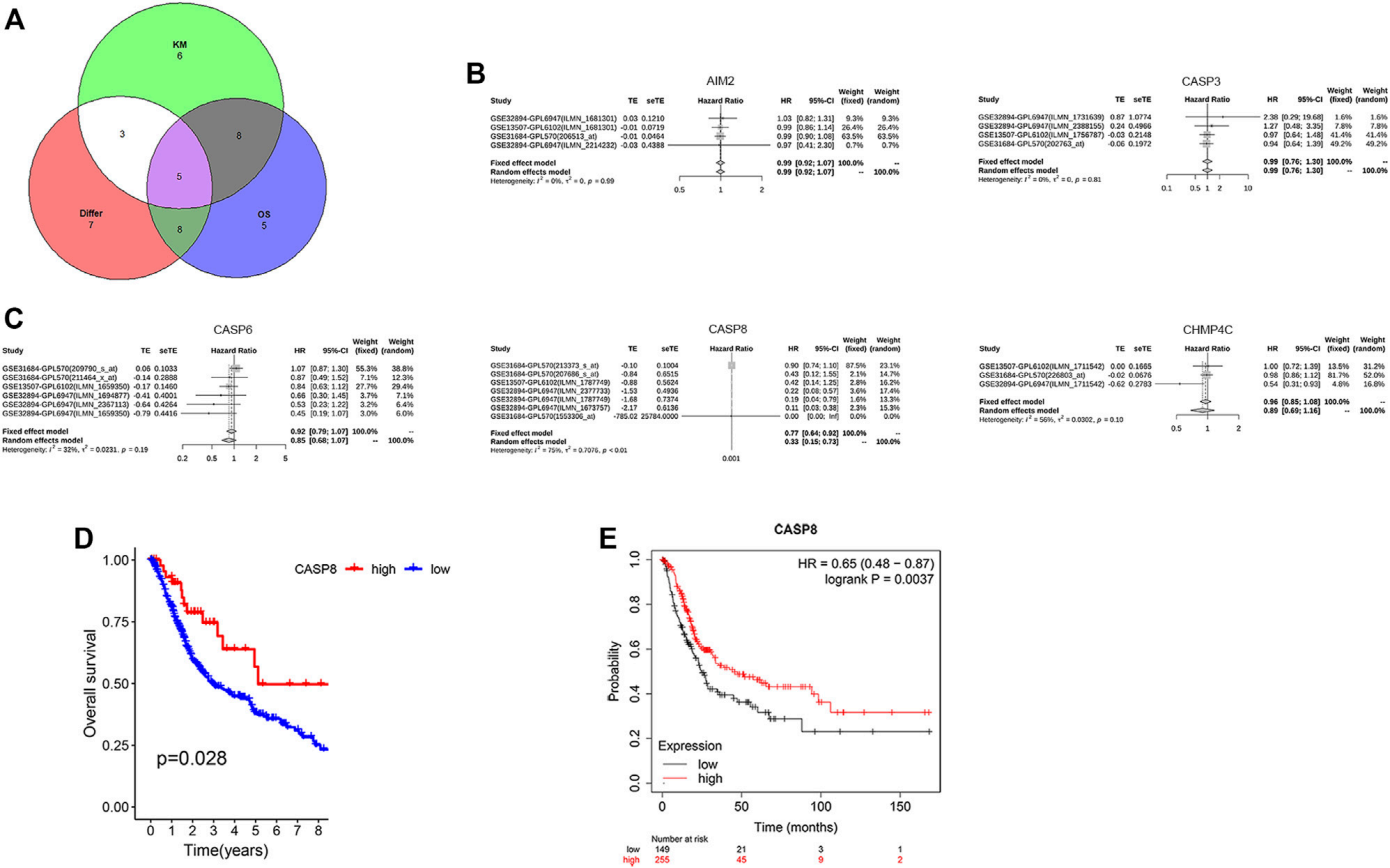
CASP8 was identified as a novel prognostic biomarker. **(A)** The intersection genes of the differentially expressed genes (DEGs) and overall survival (OS) shown by Venn diagrams. **(B,C)** Meta-survival analysis of AIM2, CASP3, CASP6, CASP8, and CHMP4C using the GENT2 database. Overall survival of CASP8 in patients with bladder cancer from **(D)** the TCGA database and GSE19423, and **(E)** the Kaplan–Meier website.

We further analyzed the relationship between the expression levels of PRGs and patient survival. Here, we identified 21 PRGs in which their expressions were related to patient survival. Further verification of the prognostic pyroptosis gene value was performed on the Kaplan–Meier website. Twenty-seven pyroptosis genes in which the expression levels were associated with patient survival were identified. Amongst these 27 PRGs, five genes (AIM2, CASP3, CASP6, CASP8, and CHMP4C) were selected by taking the intersection of the pyroptosis DEG genes and prognostic genes identified by the *R* package and website using VennDiagram package (Figure 5A). High levels of AIM2, CASP3, CASP6, CASP8, and CHMP4C expressions were associated with a superior patient survival (*p* < 0.05, for all). We further performed a meta-survival analysis based on the GENT2 database, which demonstrated that CASP8 was the only intersection gene that showed a significant difference in the expression between normal and BLCA tumor tissues (*p* < 0.01, Figures 5B,C). Moreover, patients with high expressions of CASP8 were associated with significantly better survival compared with patients with low expressions (*p* < 0.05, Figures 5D,E).

**FIGURE 6:**
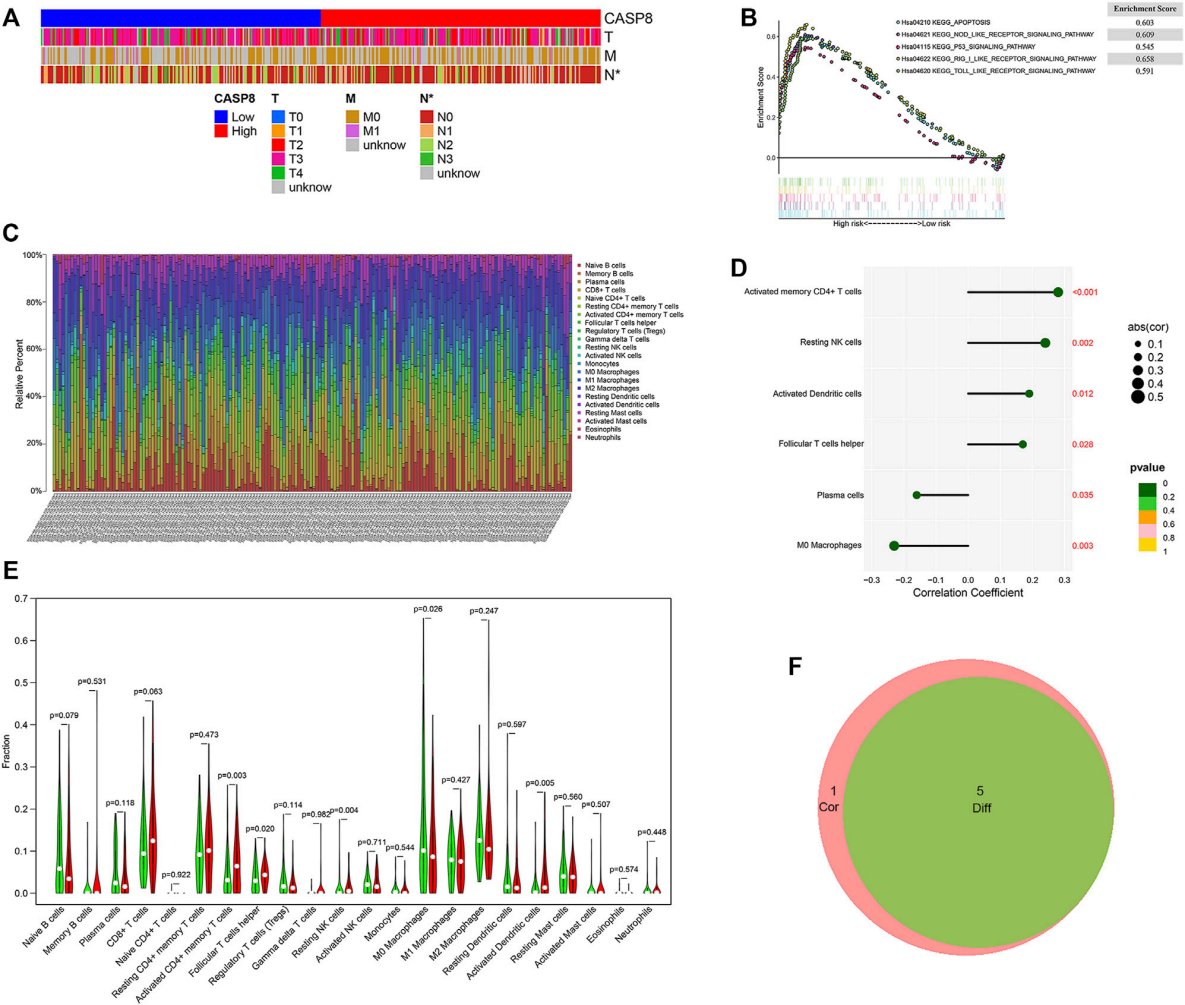
GSEA analysis of GSEA8 high expression and its relationship with clinicopathological characteristics and the proportion of tumor infiltrating cells (TICs) in bladder urothelial carcinoma (BLCA). **(A)** The relationship between CASP8 expression and clinicopathological characteristics. **(B)** The KEGG-enriched gene sets of high CASP8 expression. **(C)** Barplot displaying the ratios of the 22 types of TICs in the BLCA samples. **(D)** Lollipop plot showing the six types of TICs that had significant correlations with the CASP8 expression in BLCA samples. **(E)** Violin plot showing the expression of CASP8 in different TICs. **(F)** The Venn diagrams showing the intersection immune cells identified by difference and correlation analyses.*p < 0.05, **p < 0.01, and ***p < 0.001.

To better understanding the impact of CASP8 on tumor behavior in BLCA, we also assessed the association between the expressions of CASP8 with clinical characteristics (Figure 6A). The heatmap showed that a decreased CASP8 expression was associated with lymph node involvement in BLCA (*p* < 0.05) but was not related to the T and M scores in tumor staging.

### Gene Set Enrichment Analysis of CASP8 in BLCA

To understand the functional role of CASP8 in BLCA, GSEA was utilized to map into the KEGG pathways. The top five CASP8 overexpression gene sets were related to apoptosis, nod-like-receptor signaling pathway, toll-like-receptor signaling pathway, rig-I-like-receptor signaling pathway, and p53 signaling pathway on the basis of the cut-off criteria *p* < 0.05 (Figure 6B). The highest enriched gene set was the RIG_I_LIKE signaling pathway.

### Relationship of CASP8 With TICs in BLCA

**FIGURE 7:**
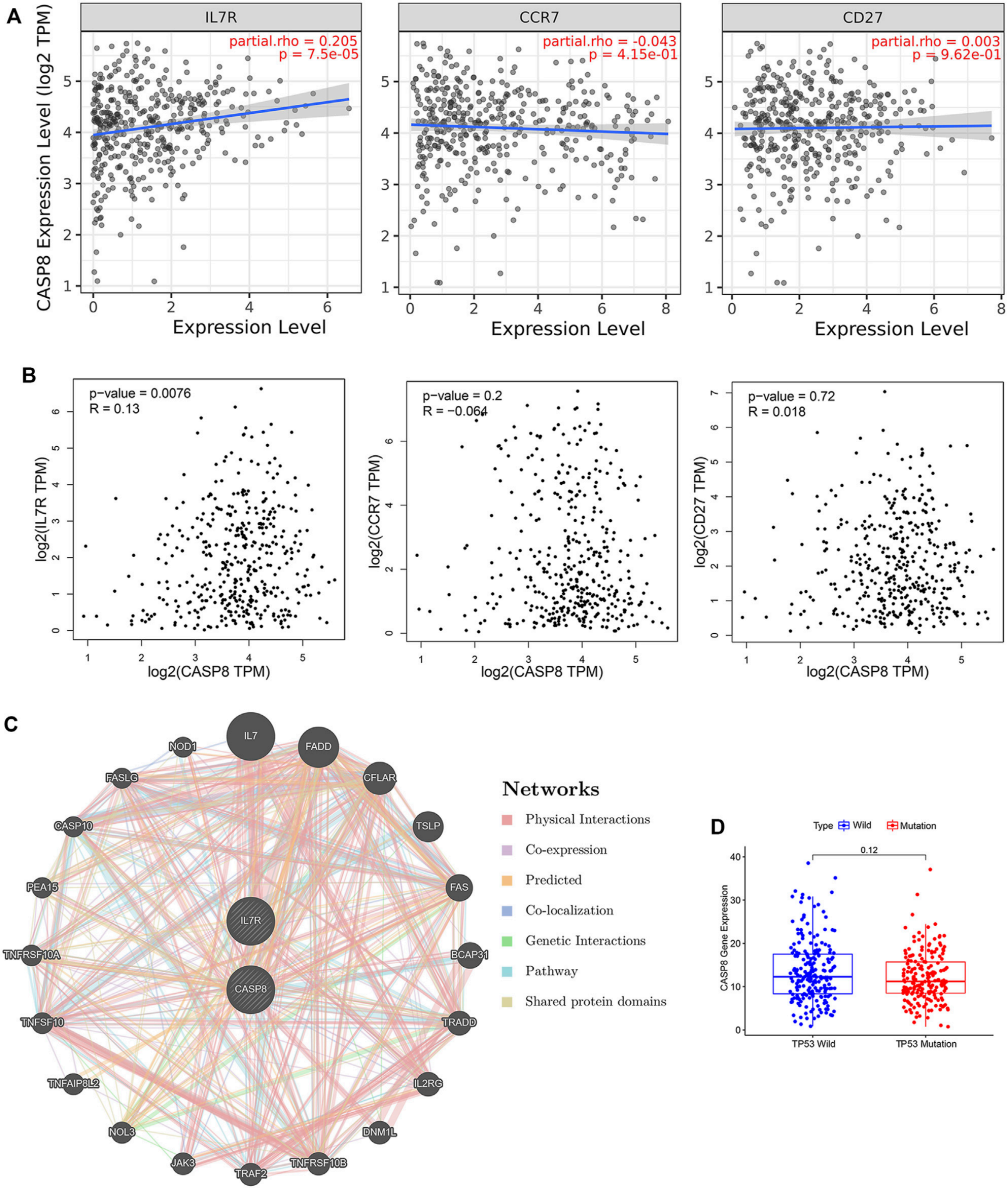
Relationship of CASP8 with cell surface markers of activated CD4+ memory T cells in bladder urothelial carcinoma (BLCA). The correlation between CASP8 and IL-7R, CCR7 and CD27 based on **(A)** the TIMER and **(B)** GEPIA databases. **(C)** Gene-to-gene network analysis of CASP8 was constructed using the Metascape website. **(D)** The expression of CASP8 in the BLCA samples with wild-type or mutations in TP53. CCR7, C-C chemokine receptor type 7; CD27, cluster of differentiation 27; IL-7R, interleukin seven receptor.

To further explore the relationship between the CASP8 expression levels and TICs, we select 22 types of immune cells related to CASP 8 using the CIBERSORT method and correlation analysis in the TCGA cohort (Figure 6C). We identified that the proportion of six populations of TICs showed significant associations with the CASP8 expression (Figure 6D). We further screened out five differential TIC fractions that showed significant difference in the CASP8 expression (Figure 6E). The intersection cells among the difference and correlation analysis identified activated CD4 memory T cells, follicular T helper cells, resting NK cells, M0 macrophages, and activated dendritic cells (Figure 6F). Activated CD4 memory T cells were used for further analysis as it showed the most significant differences in the correlation and difference tests. We further analyzed the relationship between the CASP8 expression and the cell surface markers (IL7R, CCR7, and CD27) of activated CD4 memory T cells (Figures 7A,B). The CASP8 expression showed a positive correlation with the IL7R expression. To explore the potential molecular mechanisms of CASP8 and IL7R, a gene-to-gene network was visualized using the Metascape website with help of the String database (Figure 7C), and our results showed that the IL17, FADD, CFLAR, and TSLP genes were closely linked to CASP8. TP53 mutation was associated with disease progression and an unfavorable prognosis in BLCA and is usually detected by immunohistochemistry (Lorenzo-Romero et al., 2003). We further analyzed the relationship of the CASP8 gene expression with TP 53 mutation in BLCA, which showed that samples with wild-type or mutations in TP53 had similar levels of CASP8 expression (Figure 7D).

### Correlation of CASP8 With Immune Checkpoints and Treatments in BLCA Patients

**FIGURE 8:**
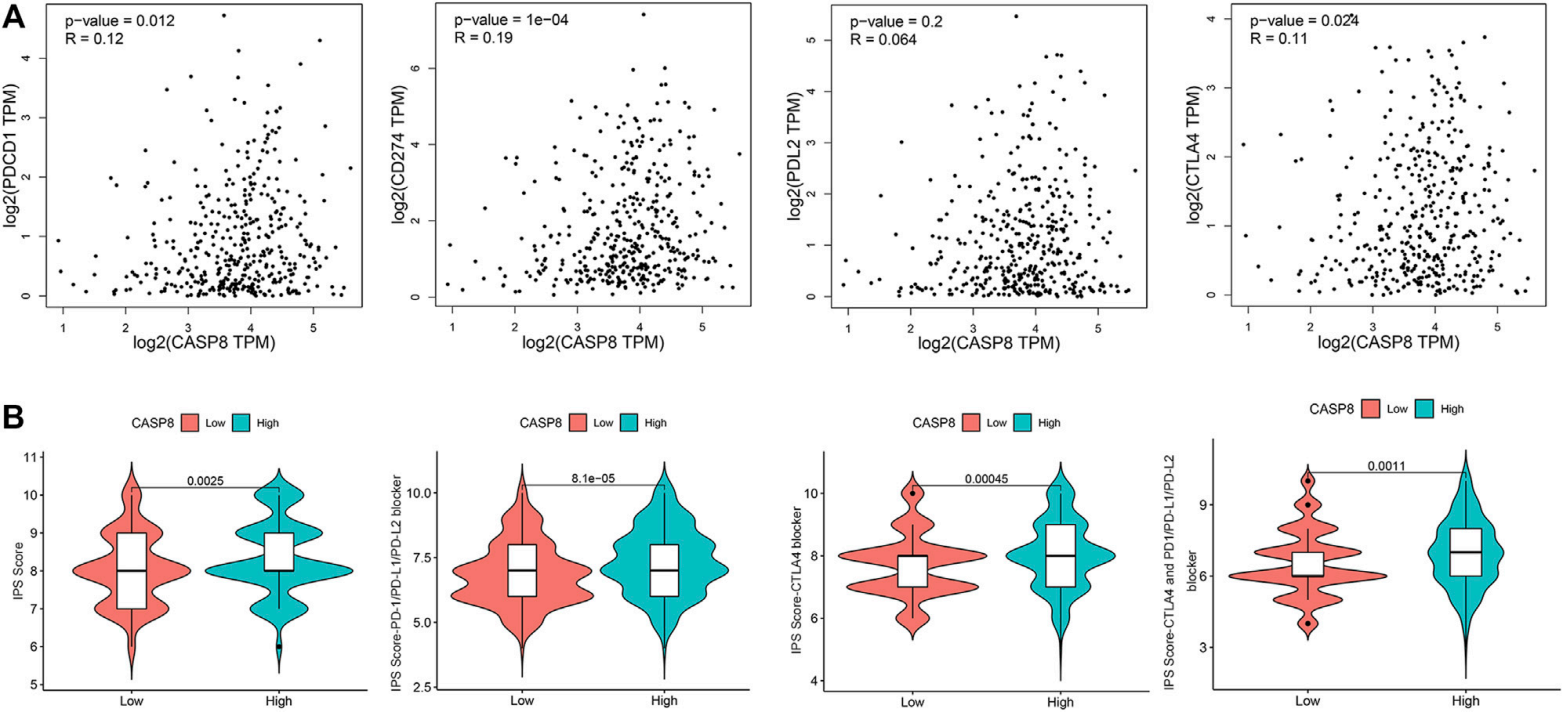
Relationship of CASP8 with immune checkpoints and response to immunotherapy in bladder urothelial carcinoma. **(A)** Correlation of the CASP8 expression with PDCD1, CD274, PDL2, and CTLA4 using the GEPIA dataset. **(B)** The impact of CASP8 expression on Immune cell Proportion Score (IPS) and the response to different immunotherapy combinations. CTLA4, cytotoxic T-lymphocyte-associated protein four; PD1, programmed cell death protein 1; PD-L1, programmed cell death ligand 1; PD-L2, programmed cell death ligand 2.

To evaluate the clinical relevance of CASP8, we explored the relationship between CASP8 expression and four immune checkpoints (CD274, CTLA4, PDL2, and PDCD1) based on the GEPIA database. CASP8 showed a positive correlation with PDCD1, CD274, and CTLA4 expressions (*p =*0.012, 1e^−04^ and 0.024, respectively, Figure 8A). However, CASP8 did not show any significant relationship with the PDL2 expression (*p* = 0.064). We further applied the Immune cell Proportion Score (IPS) to assess the efficacy of immune-checkpoint inhibitors in BLCA (Inman et al., 2017), analyzed the effect of PD1/PD-L1/PD-L2, CTLA4 and the combination of PD1/PD-L1/PD-L2, and CTLA4 treatments based on the level of the CASP8 expression. Higher expressions of CASP8 were associated with higher IPS scores for the different combinations of immunotherapy (*p* < 0.01, for all, Figure 8B). Furthermore, we also assessed the impact of the CASP8 expression on the drug sensitivity of various targeted therapies for BLCA based on the data from the GDSC database using the R package “pRRophetic.” Our results suggested that the expression level of CASP8 in BLCA could affect the drug sensitivities (IC50) of FR-180204 (an ERK inhibitor), LY317615 (a PKCβ inhibitor), JNK VIII inhibitor, gefitinib (EGFR inhibitor), SNX-2112 (HSP inhibitor), and lapatinib (an EGFR/HER2 tyrosine kinase inhibitor) (*p* < 0.001, for all) (Supplementary Figure S1).

## Discussion

Accumulating evidence has suggested the crucial roles of pyroptosis in tumor immune infiltration (Loveless et al., 2021), but its pathogenic significance in BLCA remains undefined. Our study was the first to use a bioinformatics approach to elucidate the relationship between PRGs, clinical outcomes, and the response to immune-checkpoint inhibitors (ICPIs) in BLCA. Our results suggested that distinct patterns of PRGs were predictive of patient survival and CASP8 expression was associated with patient prognosis, activated CD4 memory T cells within TICs, and therapeutic response to ICPIs.

Our present findings showed that aberrant expressions of PRGs were related to BLCA, with missense mutations and SNPs being the most common genetic abnormalities. In this study, we identified a panel of PRGs in BLCA, including a number of genes in the caspase family. Previous studies have reported that caspase families including CASP-1, 3, 4, 5, and 6 could induce pyroptosis (Jiang et al., 2019; Ruan et al., 2020) and have crucial roles in tumourigenesis and immunological disorders (Zheng and Li, 2020). The role of caspases in tumor biology is highly complex. Our current data suggested that high levels of CASP3, CASP6, CASP8, and CHMP4C expressions were associated with improved patient survival. It remains speculative whether increased expressions of CASP3, CASP6, and CASP8 may be associated with enhanced pyroptosis and cell death, thereby reducing the cancer’s aggressiveness. Indeed, CASP8 is an important switch for the activation of apoptosis and pyroptosis (Schwarzer et al., 2020), and shows pleiotropic effects in cancer development (Schwarzer et al., 2020). Our results indicated that CASP8 plays a pivotal role in BLCA and CASP8 and was overexpressed in BLCA patients compared with normal individuals. Up-regulated CASP8 expressions were observed in other urogenital cancers including renal (de Martino et al., 2013) neoplasms. Data regarding CASP8 expression in BLCA are conflicting. One study in China showed that the caspase-8 rs3834129 genotype was associated with an increased risk of BLCA, while another study in Taiwan did not show the same correlation (Wang et al., 2009; Chen et al., 2019). While CASP8 expression was increased in BLCA patients compared with healthy individuals, our data also indicated that among BLCA patients, a high expression of CASP8 was associated with better prognosis. Reduced CASP8 expression has also been linked to certain neoplastic behaviors such as metastasis (Kordi Tamandani et al., 2009; Zhu et al., 2010), for instance in a breast cancer cell line (MDA-MB-231), decreased CASP8 expression was associated with increased tumor migration and invasion [36], and therefore, it remains possible that an up-regulation of CASP8 may confer a survival advantage. Previous studies have also reported an increased CASP8 expression in human bladder cells after chemotherapy (Ahmed et al., 2015). Here, we also found that a high CASP8 expression was most enriched in the RIG_I_LIKE pathway, which might have anti-cancer functions in breast cancer, prostate cancer, and ovarian cancer cell lines, and colorectal cancer human cancer tissues (Wu et al., 2017). Similar results were found in cervical (Ekonomopoulou et al., 2011) and renal caners (de Martino et al., 2013). On the contrary, the down-regulation and low-activity of CASP8 had been reported in various solid organ tumors including liver cancer, small cell lung cancer, gastric adenocarcinoma, and brain tumors (Stupack, 2013). Other investigators have reported that increased expressions of other caspase families could accelerate tumor onset. For instance, CASP3 knockout in colon cancer cells showed less invasion and metastases (Zhou et al., 2018), while the up-regulation of CASP5 was observed in clear cell renal cell carcinoma (Zhang et al., 2021).

Our analysis also identified a cluster of pyroptosis-related DEGs in BLCA, which include GSDMB, BAX, TP63, CHMP2A, and GPX4. These findings were in line with one recent bioinformatics study on PRGs in BLCA (Fu and Wang, 2022). To enhance the clinical relevance of our data, we devised a PRG risk score that can predict the survival in BLCA patients. Our proposed prognostic risk model, which incorporated patient demographics, tumor characteristics, and the DEGclusters, showed promising performance in predicting the OS at 1, 3, and 5 years in BLCA patients. Compared to previous predictive models, our BLCA pyroptosis-related prognostic risk model was developed with seven independent prognostic genes based on the expression of 1,792 subgroup-related DEGs and thus showed an improved performance (Chen et al., 2021).

As pyroptosis represents a form of programmed cell death triggered by inflammasomes in response to immune activation, it is pertinent to investigate the relationship between pyroptosis and immune cells in BLCA. Different immune reactive cells and related cytokine pathways contribute to tumor growth and development (Ten Hacken and Burger, 2016). Our data suggested that an aberrant expression of CASP8 was associated with the abnormal proportions of distinct immune cell populations including activated CD4 memory T cells, follicular T helper cells, resting NK cells, M0 macrophages, and activated dendritic cells. Among these important immune cell types, we found that CASP8 was most related to activated CD4 memory T cells. This was also corroborated by the significant correlation between CASP8 and IL7R expressions—a key cell surface marker of activated CD4 memory T cells. In the context of cancer immunology, CD4 memory T cells play an instrumental role in TME and increased infiltration was detected in colorectal cancer and triple-negative breast cancer (Deng et al., 2019; Ge et al., 2019). A high proportion of activated CD4 memory T cells was found in non-small cell lung cancer (Oja et al., 2018) and in renal cell carcinoma after radiation therapy (Seung et al., 2012). Indeed, the role of CASP8 in immune regulation has also been reported in other studies. For instance, deletion of CASP 8 in T cells of mice resulted in the reduction of T lymphocytes and T cell-associated immunodeficiency (Salmena et al., 2003). Moreover, CASP8 could inhibit NK cell proliferation in a murine model of cytomegalovirus infection (Feng et al., 2019). In ovarian cancer, caspase-8 can modulate B and T lymphocyte activation, as well as macrophage differentiation and polarization (Kostova et al., 2021).

Recognizing the strong association between CASP8 and activated CD4 memory T cells, we also investigated the impact of CASP8 expression on immune checkpoint expression and response to ICPIs. Commonly used ICPIs include anti-PD1, anti-PDL1, and anti-CTLA4 blocking antibodies (Marin-Acevedo et al., 2021). These ICPIs are increasingly popular in different cancers including BLCA due to their promising treatment efficacy and tolerability (Ott et al., 2020). In this context, pembrolizumab (a monoclonal antibody against PD1 receptor) is a useful treatment for platinum-ineligible metastatic bladder cancer that shows an increased PD-L1 expression (>10%) and is also an important second-line treatment for advanced bladder cancer (Bellmunt et al., 2017; Balar et al., 2021). Nivolumab (an anti-PD1 monoclonal antibody) is also indicated in bladder cancer with a PDL1 expression >1% in the post-operative adjuvant setting (Bajorin et al., 2021). In this study, we found a positive correlation between CASP8 and various key immune checkpoints including PDCD1 (programmed cell death 1 or PD-1), CD274 (programmed cell death ligand 1 or PD-L1), and CTLA4 (cytotoxic T-lymphocyte-associated protein 4) expressions. Our findings also suggested that an increased CASP8 expression was associated with better treatment efficacy of different combinations of ICPIs. Whether the assessment of the CASP8 expression may add value to the current practice of immunohistochemical staining in choosing BLCA patients for immunotherapy remain to be investigated. Our study also demonstrated that the expression level of CASP8 in BLCA was related to the drug sensitivity of various targeted therapies including FR-180204 (an ERK inhibitor), LY317615 (a PKCβ inhibitor), JNK VIII inhibitor, gefitinib (EGFR inhibitor), SNX-2112 (HSP inhibitor), and lapatinib (an EGFR/HER2 tyrosine kinase inhibitor). These results have the potential to guide the use of therapeutics in patients with BLCA in the era of personalized medicine. Indeed, EGFR/HER2 protein overexpressions have been reported in some bladder cancer tissues and these are associated with more aggressive diseases. Preliminary clinical evidence suggested that patients with HER2 amplification responded to pan-kinase inhibitor afatinib (Choudhury et al., 2016). New antibody-drug conjugate RC48-ADC also demonstrated an encouraging objective response rate of 60% in HER2+ urothelial cancer (Sheng et al., 2021). These observations suggest that the study of CASP8 as an exploratory biomarker for bladder cancer treatment may be worthwhile. Furthermore, the relationship between CASP8, patient survival, TICs, and ICPIs as demonstrated by our data may open new avenues for treatment of BLCA, making CASP8 an attractive therapeutic target (Stupack, 2013). For instance, one important way to activate CASP8-related apoptosis is by engaging the tumor necrosis factor-related apoptosis-inducing ligand (TRAIL; TNFSF10)-receptor, which could mediate CASP8-related apoptosis (Ning et al., 2016).

One important limitation of this study was that the results were generated from the secondary analysis of data obtained from public domains. Notwithstanding that, the current analysis has included data from different major gene websites, some of which contain detailed patient characteristics and outcomes. Future translational and clinical studies will be worthwhile to validate the findings of our study and further elucidate the underlying molecular mechanism and relationship with the tumor microenvironment in BLCA.

## Conclusion

Our data suggest that PRGs, especially CASP8, showed strong associations with patient prognosis and TICs in BLCA. These results are clinically useful for prognostication and selection of treatments in BLCA patients.

## Data Availability

The datasets presented in this study can be found in online repositories. The names of the repository/repositories and accession number(s) can be found in the article/Supplementary Material.

## Supplementary Material

Supplementary Figure S1 The relationship between CASP8 expression and drug sensitivity (IC50) of different pharmacological compounds in bladder urothelial carcinoma.

## Abbreviations

BLCA, bladder urothelial carcinoma; CASP8, caspase-8; CTLA4, cytotoxic T-lymphocyte-associated protein 4; GEO, gene expression omnibus; GENT2, gene expression database of normal and tumor tissues; GSEA, gene set enrichment analysis; GEPIA, gene expression profiling interactive analysis; GDSC, genomics of drug sensitivity in cancer; IPS, immune cell proportion score; ICPIs, immune-checkpoint inhibitors; KEGG, kyoto encyclopedia of genes and genomes; PDCD1, programmed cell death 1; CD274 or PD-L1, programmed cell death ligand 1; TCGA, the cancer genome atlas; TIMER, tumor immune estimation resource; TCIA, the cancer immunome atlas.
