# Supplementary figures and images for: The Relationship of Pyroptosis-Related Genes, Patient Outcomes, and Tumor-Infiltrating Cells in Bladder Urothelial Carcinoma (BLCA)
Source: Front Pharmacol. 2022 Jul 19;13:930951. doi: 10.3389/fphar.2022.930951 (PMC9343957; doi:10.3389/fphar.2022.930951)

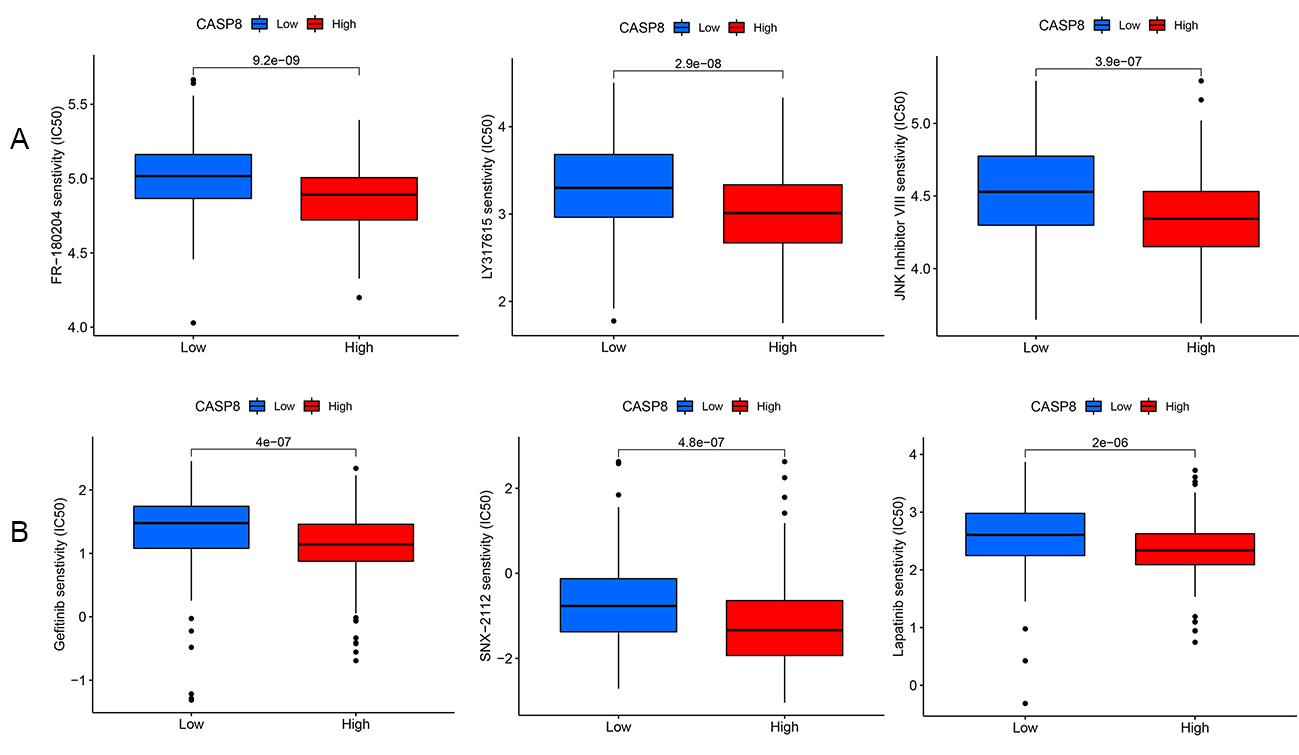

Supplement: Supplementary file 1 [file Image1.TIF]
